# Association of Serum Vitamin D Levels With the Severity of Chronic Heart Failure: A Retrospective Study

**DOI:** 10.7759/cureus.114248

**Published:** 2026-08-10

**Authors:** Farah Nazir, Tayyaba Shabbir, Semil Saleem, Behroze Khalid, Saad Binliaquat, Sadia Yousaf, Somia Bibi

**Affiliations:** 1 Internal Medicine, Jinnah Hospital Lahore, Lahore, PAK; 2 Internal Medicine, King Edward Medical University, Lahore, PAK; 3 Internal Medicine, Rawalpinidi Medical University, Rawalpinidi, PAK

**Keywords:** association, chronic, clinical, failure, heart, left ventricular ejection fraction, outcomes, serum, severity, vitamin d

## Abstract

Background

Chronic heart failure (CHF) is a leading cause of morbidity and mortality worldwide. Although vitamin D insufficiency has been implicated in its pathophysiology, its association with CHF severity remains uncertain. Therefore, this study primarily evaluated the association between serum vitamin D levels and CHF severity in hospitalized adults. Secondary analyses assessed the relationship with left ventricular ejection fraction, factors associated with severe CHF, and selected in-hospital outcomes.

Methods

This retrospective study included 276 patients with chronic heart failure managed at a tertiary care hospital in Pakistan. Demographic, clinical, laboratory, and echocardiographic data were retrieved from patients’ medical records. Based on the New York Heart Association (NYHA) functional classification, patients were categorized into severe (Class III-IV) and non-severe (Class I-II) heart failure groups. Statistical analyses were carried out. Continuous variables were compared using the independent-samples t-test, categorical variables using the chi-square test, and the association between serum vitamin D levels and left ventricular ejection fraction (LVEF) was assessed using Pearson correlation analysis. Univariate and multivariable logistic regression analyses were performed to identify independent predictors of severe chronic heart failure.

Results

Among the 276 patients, 168 (60.86%) had severe CHF and 108 (39.14%) had non-severe CHF. The mean ± standard deviation (SD) age was 63.52 ± 11.24 years, the mean serum vitamin D concentration was 21.16 ± 8.12 ng/mL, and the mean LVEF was 38.54 ± 9.82%. Overall, 208 (75.36%) patients had vitamin D insufficiency. Patients with severe CHF had significantly lower serum vitamin D levels and LVEF than those with non-severe CHF (p<0.001). Serum vitamin D levels demonstrated a moderate positive correlation with LVEF (r=0.48, 95% CI: 0.38-0.57; p<0.001). After multivariable adjustment, vitamin D insufficiency was the strongest independent predictor of severe CHF (adjusted odds ratio [AOR] 3.36, 95% CI: 1.88-6.02; p<0.001), while previous ischemic heart disease (AOR 1.81, 95% CI: 1.01-3.23; p=0.045) and diabetes mellitus (AOR 1.92, 95% CI: 1.08-3.42; p=0.026) also remained significant independent predictors. Patients with vitamin D insufficiency had significantly longer hospital stays, higher coronary care unit admission rates, and greater in-hospital mortality than those with normal vitamin D levels (p<0.05).

Conclusions

Lower serum vitamin D levels were significantly associated with increased chronic heart failure severity. Vitamin D insufficiency was highly prevalent and emerged as the strongest independent predictor of severe heart failure, while diabetes mellitus and previous ischemic heart disease were also independent predictors. Furthermore, vitamin D insufficiency was associated with poorer in-hospital outcomes. Assessment of vitamin D status may complement conventional clinical and echocardiographic evaluation for risk stratification in patients with chronic heart failure.

## Introduction

Chronic heart failure (CHF) is a major global cardiovascular health challenge and a leading cause of morbidity and mortality worldwide. It is a complex clinical syndrome caused by structural or functional cardiac abnormalities that impair the heart's ability to maintain adequate cardiac output, resulting in symptoms such as exertional dyspnea, fatigue, reduced exercise tolerance, and fluid retention [1,2]. Heart failure severity is commonly assessed using the New York Heart Association (NYHA) functional classification, which categorizes patients according to the degree of functional limitation imposed by their symptoms during physical activity [3,4]. The prevalence of CHF continues to increase due to population aging, longer life expectancy, and improved survival following cardiovascular diseases, with an estimated 37.7 million people affected globally [5]. Despite substantial advances in guideline-directed medical therapy, CHF remains associated with poor long-term outcomes, with 5-year mortality rates approaching 60-70% [6]. Furthermore, CHF imposes a substantial economic burden on healthcare systems because of frequent hospitalizations, prolonged treatment, and reduced quality of life [1,2].

The burden of CHF is further exacerbated by the increasing prevalence of cardiovascular risk factors, including coronary artery disease, hypertension, diabetes mellitus, and obesity. This challenge is particularly pronounced in low- and middle-income countries such as Pakistan, where limited healthcare resources, delayed diagnosis, and restricted access to specialized cardiac care contribute to poorer clinical outcomes. Consequently, identifying potentially modifiable factors associated with heart failure severity remains essential for improving risk stratification and patient management [1,2,5,7].

Vitamin D deficiency has emerged as a potential contributor to the progression and severity of CHF. Several observational studies have reported an inverse association between serum vitamin D concentrations and heart failure severity, with lower vitamin D levels associated with impaired cardiac function, greater symptom burden, and poorer clinical outcomes [8-11]. Beyond its established role in calcium homeostasis and bone metabolism, vitamin D contributes to cardiovascular function through receptors expressed in cardiomyocytes, vascular smooth muscle cells, and endothelial cells. Vitamin D deficiency may promote the progression of chronic heart failure by activating the renin-angiotensin-aldosterone system, increasing oxidative stress and systemic inflammation, and promoting myocardial fibrosis and adverse ventricular remodeling, ultimately leading to impaired cardiac function and worsening heart failure severity [12-15].

Vitamin D deficiency is highly prevalent in Pakistan, affecting a large proportion of the population despite adequate sunlight availability. Contributing factors include inadequate dietary intake, limited sun exposure, urban lifestyles, and poor awareness regarding vitamin D supplementation [16,17]. The potential role of vitamin D in chronic heart failure remains uncertain. Although several studies have reported associations between lower serum vitamin D levels and greater heart failure severity and adverse clinical outcomes [8,18,19], other studies have failed to demonstrate a significant relationship [20,21]. Similarly, clinical trials investigating vitamin D supplementation in CHF have yielded conflicting results, with some reporting improvements in cardiac parameters, including left ventricular ejection fraction [22], while others showed no significant clinical benefit [20,23]. Moreover, most available evidence originates from high-income countries, limiting its applicability to resource-limited settings such as Pakistan. Therefore, further research is needed to better understand the association between vitamin D status and CHF severity in these populations.

Therefore, this study primarily aimed to evaluate the association between serum 25-hydroxyvitamin D levels and chronic heart failure severity among hospitalized adult patients in Pakistan, as determined by the New York Heart Association functional classification. Secondary objectives were to assess the relationship between vitamin D levels and left ventricular ejection fraction and identify factors independently associated with severe heart failure. In-hospital outcomes were explored as a secondary analysis. These findings may help further characterize the clinical relevance of vitamin D status in chronic heart failure and provide a basis for future prospective studies evaluating its potential role in risk assessment and management.

## Materials and methods

Study design and study population

This retrospective study was conducted at Benazir Bhutto Hospital, Rawalpindi, Pakistan. Out of 389 medical records screened for eligibility, 276 consecutive adult patients with documented chronic heart failure who fulfilled the predefined inclusion and exclusion criteria and were managed as hospital patients between June 2022 and June 2025 were included in the analysis. The specific indication for hospitalization was not consistently available due to the retrospective nature of the study. Hospital outcomes, including length of stay, CCU admission, and in-hospital mortality, were recorded where available. Ethical approval was obtained before data collection (Approval No. BBH.ERB.282/312), and patient confidentiality was maintained throughout the study.

Inclusion and exclusion criteria

**Figure 1:**
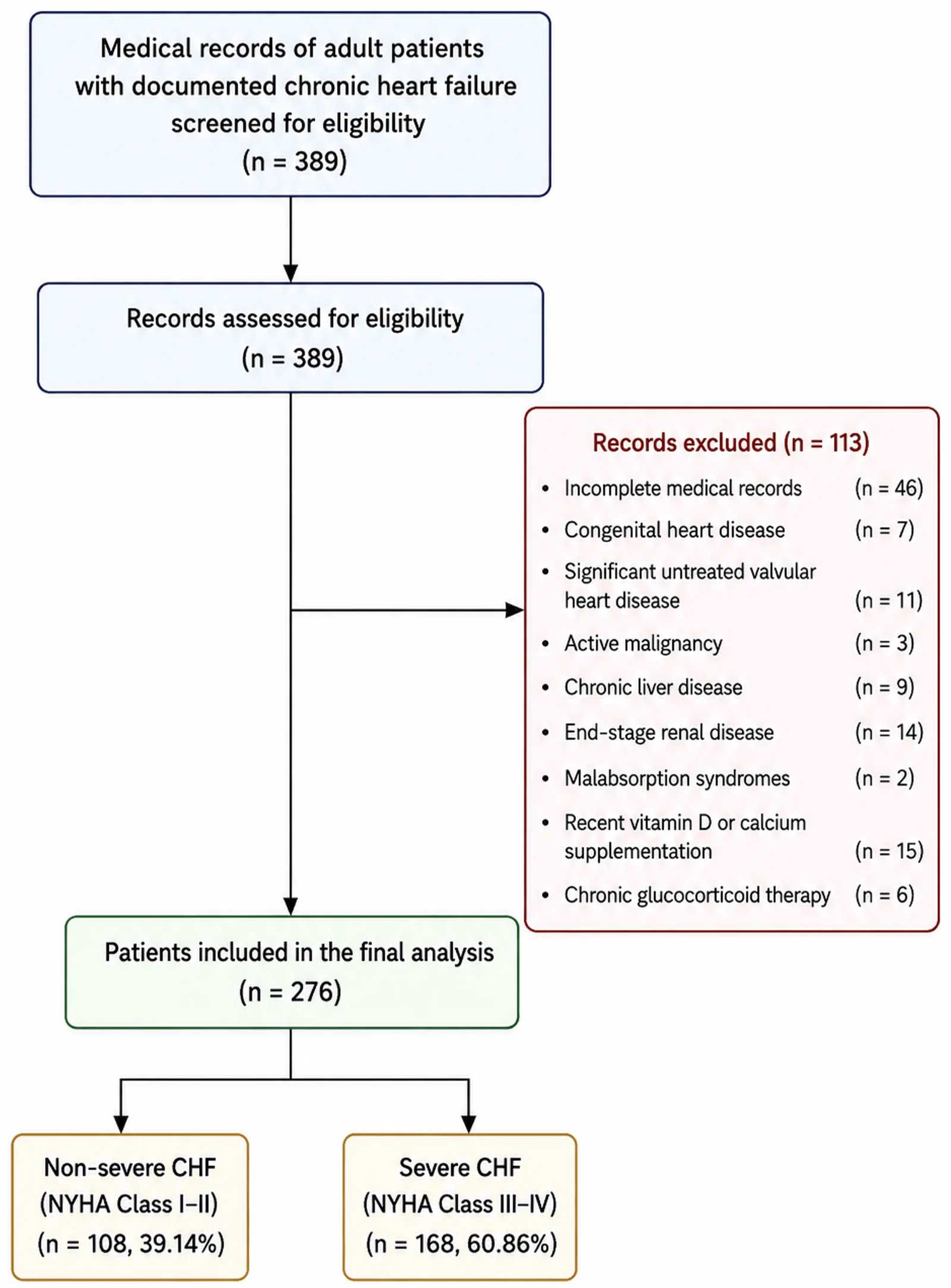
Participant flow diagram of patient screening, eligibility, exclusions, and final inclusion CHF: chronic heart failure; NYHA: New York Heart Association

Patients aged ≥18 years with a confirmed diagnosis of chronic heart failure based on clinical assessment and echocardiographic findings were included. Only patients with complete medical records, including demographic information, laboratory investigations, serum vitamin D levels, and left ventricular ejection fraction assessed by transthoracic echocardiography, were eligible for analysis. Patients with congenital heart disease, significant untreated valvular heart disease, active malignancy, chronic liver disease, end-stage renal disease, disorders affecting vitamin D metabolism, malabsorption syndromes, recent vitamin D or calcium supplementation, or chronic glucocorticoid therapy were excluded. Figure 1, a study flow diagram, shows the inclusion and exclusion criteria section to improve the transparency of participant selection and study reporting.

Study objectives

The primary objective of this study was to evaluate the association between serum vitamin D levels and the severity of chronic heart failure, as determined by the New York Heart Association functional classification, among hospitalized adult patients with chronic heart failure. Secondary objectives were to compare serum vitamin D levels between patients with severe and non-severe chronic heart failure, assess the relationship between serum vitamin D levels and left ventricular ejection fraction, and identify clinical factors independently associated with severe chronic heart failure. In addition, the association between vitamin D status and selected in-hospital clinical outcomes, including length of hospital stay, coronary care unit (CCU) admission, and in-hospital mortality, was explored as a secondary analysis.

Evaluation of study variables

Chronic heart failure was diagnosed according to the American Heart Association (AHA), American College of Cardiology (ACC), and Heart Failure Society of America (HFSA) guidelines, based on documented clinical symptoms, physical examination findings, and supportive echocardiographic evidence [8,24]. Heart failure severity was assessed using the New York Heart Association functional classification. For analysis, patients were categorized into non-severe heart failure (NYHA Class I-II) and severe heart failure (NYHA Class III-IV) groups [3,4]. Left ventricular ejection fraction was measured using transthoracic echocardiography. Serum vitamin D status was measured during hospitalization using a chemiluminescence immunoassay (CLIA). Vitamin D status was categorized as normal (≥30 ng/mL) or insufficient (<30 ng/mL), based on the threshold used in the cited literature [10,25].

Data collection

Data were collected using a self-designed structured data collection proforma comprising two sections. The first section included demographic and clinical variables, including age, sex, history of ischemic heart disease, diabetes mellitus, hypertension, dyslipidemia, smoking status, and other relevant clinical characteristics, together with clinical outcomes including duration of hospital stay, coronary care unit (CCU) admission, and in-hospital mortality. The second section included laboratory and echocardiographic findings, including serum vitamin D levels, relevant laboratory parameters, left ventricular ejection fraction, and NYHA functional classification.

Data analysis

Statistical analysis was performed using IBM SPSS Statistics for Windows, Version 25 (IBM Corp., Armonk, USA). Continuous variables were expressed as mean ± standard deviation, whereas categorical variables were presented as frequencies and percentages. Normality of continuous variables was assessed using the Shapiro-Wilk test. Differences between study groups were evaluated using the independent-samples t-test for continuous variables and the chi-square test for categorical variables. Pearson correlation analysis was used to assess the relationship between serum vitamin D levels and left ventricular ejection fraction. Multivariable logistic regression analysis was performed to identify factors independently associated with severe chronic heart failure. Variables with clinical relevance or statistical significance on univariate analysis (p≤0.05) were included in the model. A p-value <0.05 was considered statistically significant.

## Results

A total of 276 patients with chronic heart failure were included in the final analysis. Of these, 168 (60.86%) were categorized as having severe heart failure, whereas 108 (39.14%) had non-severe heart failure. The overall mean ± standard deviation (SD) age was 63.52 ± 11.24 years, the mean serum vitamin D concentration was 21.16 ± 8.12 ng/mL, and the mean left ventricular ejection fraction was 38.54 ± 9.82%. Overall, 208 (75.36%) patients had vitamin D insufficiency, whereas 68 (24.64%) had normal vitamin D levels.

Table 1 shows that patients with severe heart failure were significantly older and had lower serum vitamin D levels than those with non-severe heart failure. The mean serum vitamin D concentration was significantly lower among patients with severe heart failure compared with the patients with non-severe heart failure (p <0.001). Likewise, mean LVEF was significantly lower in the severe heart failure group than in the non-severe group (p <0.001). Previous ischemic heart disease, diabetes mellitus, hypertension, and physical inactivity were significantly more common among patients with severe heart failure, whereas no significant differences were observed for gender, dyslipidemia, or smoking status.

**Table 1:** Baseline demographic and clinical characteristics according to heart failure severity NYHA: New York Heart Association; SD: standard deviation; ng/mL: nanograms per milliliter; LVEF: left ventricular ejection fraction; CHF: chronic heart failure

Variable (N=276)		Value of variables	Study groups based on CHF severity (NYHA)		Independent t-test/ Chi-square test	
					Test Statistics	
			Severe CHF (n=168, 60.86%)	Non-severe CHF (n=108, 39.14%)	t-value for Independent-test/ χ²-value for Chi-Square test	p-value
Age (years), mean ± SD		63.52 ± 11.24	65.24 ± 10.62	60.82 ± 11.50	t = 2.94	0.004
Serum vitamin D (ng/mL), mean ± SD		21.16 ± 8.12	17.92 ± 6.55	25.84 ± 7.32	t = 9.35	<0.001
LVEF (%), mean ± SD		38.54 ± 9.82	33.44 ± 7.26	46.58 ± 7.82	t = 14.17	<0.001
Age category	<65 years, n (%)	140 (50.72)	74 (44.07)	66 (61.10)	χ² = 5.65	0.018
	≥65 years, n (%)	136 (49.28)	94 (55.93)	42 (38.90)		
Gender	Male, n (%)	167 (60.50)	104 (61.90)	63 (58.34)	χ² = 0.32	0.570
	Female, n (%)	109 (39.50)	64 (38.10)	45 (41.66)		
Vitamin D status	Insufficiency n (%)	208 (75.36)	144 (85.71)	64 (59.25)	χ² ≈ 22.8	<0.001
	Normal n (%)	68 (24.64)	24 (14.29)	44 (40.75)		
Previous ischemic heart disease	Yes, n (%)	170 (61.59)	112 (66.66)	58 (53.70)	χ² = 4.44	0.034
	No, n (%)	106 (38.41)	56 (33.34)	50 (46.30)		
Diabetes mellitus	Yes, n (%)	139 (50.36)	96 (57.14)	43 (39.81)	χ² = 7.53	0.005
	No, n (%)	137 (49.64)	72 (42.86)	65 (60.19)		
Hypertension	Yes, n (%)	182 (65.94)	119 (70.84)	63 (58.33)	χ² = 4.30	0.041
	No, n (%)	94 (34.06)	49 (29.16)	45 (41.67)		
Dyslipidemia	Yes, n (%)	116 (42.02)	77 (45.84)	39 (36.11)	χ² = 2.42	0.126
	No, n (%)	160 (57.98)	91 (54.16)	69 (63.89)		
Smoking status	Yes, n (%)	94 (34.07)	63 (37.50)	31 (28.70)	χ² = 2.07	0.154
	No, n (%)	182 (65.93)	105 (62.50)	77 (71.30)		
Physical inactivity	Yes, n (%)	132 (47.82)	90 (53.56)	42 (38.88)	χ² = 5.24	0.024
	No, n (%)	144 (52.18)	78 (46.44)	66 (61.12)		

Table 2 demonstrates a moderate positive correlation between serum vitamin D levels and left ventricular ejection fraction, indicating that higher serum vitamin D concentrations were associated with better left ventricular systolic function.

**Table 2:** Correlation between serum vitamin D levels and left ventricular ejection fraction. LVEF: left ventricular ejection fraction; CHF: chronic heart failure; SD: standard deviation; ng/mL: nanogram per milliliter

Variables of Patients with CHF (N=276)	Study Groups based on CHF severity		Independent t-test		Pearson’s Correlation		
			Test Statistics		Test Statistics		
	Severe CHF Group	Non-severe CHF Group	t-value	p-value	Correlation Coefficient (r)	95% CI	p-value
LVEF (%), mean ± SD	33.44 ± 7.26	46.58 ± 7.82	14.17	<0.001	0.48	0.38–0.57	<0.001
Serum Vitamin D Level (ng/mL) (Means ± SD)	17.92 ± 6.55	25.84 ± 7.32	9.35	<0.001			

Table 3 demonstrates that, in the univariate analysis, vitamin D insufficiency, age ≥65 years, previous ischemic heart disease, diabetes mellitus, hypertension, and physical inactivity were significantly associated with severe chronic heart failure. After multivariable adjustment, vitamin D insufficiency remained independently associated with severe heart failure, while previous ischemic heart disease and diabetes mellitus also remained significantly associated with severe CHF.

**Table 3:** Univariate and multivariable logistic regression analysis for predictors of severe chronic heart failure (NYHA Class III–IV) NYHA: New York Heart Association

Variable	Crude OR	95% CI	p-value	Adjusted OR	95% CI	p-value
Age ≥65 years	1.82	(1.11–2.98)	0.018	1.43	(0.82–2.49)	0.206
Male sex	1.14	(0.69–1.89)	0.570	1.09	(0.64–1.87)	0.742
Vitamin D insufficiency	4.28	(2.49–7.37)	<0.001	3.36	(1.88–6.02)	<0.001
Previous Ischemic heart disease	1.72	(1.04–2.86)	0.034	1.81	(1.01–3.23)	0.045
Diabetes mellitus	2.02	(1.23–3.33)	0.005	1.92	(1.08–3.42)	0.026
Hypertension	1.74	(1.03–2.94)	0.041	1.29	(0.73–2.28)	0.376
Dyslipidemia	1.49	(0.89–2.48)	0.126	1.18	(0.67–2.07)	0.567
Smoking	1.52	(0.87–2.56)	0.154	1.28	(0.72–2.27)	0.399
Physical inactivity	1.81	(1.08–3.03)	0.024	1.58	(0.89–2.81)	0.118

Table 4 summarizes in-hospital clinical outcomes by vitamin D status. Patients with vitamin D insufficiency had significantly poorer outcomes, including longer hospital stays, higher CCU admission rates, and higher in-hospital mortality compared with those with normal vitamin D levels.

**Table 4:** Association between vitamin D status and In-hospital clinical outcomes among patients with chronic heart failure SD: standard deviation; CCU: coronary care unit

Clinical outcome	Insufficient vitamin D level (n=208)	Normal vitamin D level (n=68)	p-value
Hospital stay (days), mean ± SD	7.50 ± 2.84	5.30 ± 2.10	<0.001
CCU admission, n (%)	62 (29.81)	9 (13.20)	0.006
In-hospital mortality, n (%)	37 (17.80)	5 (7.35)	0.030

## Discussion

Chronic heart failure remains a major cause of morbidity, mortality, and recurrent hospitalization worldwide. Identifying readily available biomarkers to improve clinical assessment is therefore of considerable clinical importance [1,2,7]. The present study demonstrated that lower serum vitamin D levels were significantly associated with greater CHF severity, reduced left ventricular ejection fraction, and poorer in-hospital outcomes. Moreover, vitamin D insufficiency remained independently associated with severe CHF after adjustment for the variables included in the multivariable model. These findings suggest that assessment of serum vitamin D may provide additional clinical information alongside conventional clinical and echocardiographic evaluation, with potential implications for risk stratification and management of patients with CHF, particularly in resource-limited settings [9,10].

In the present study, 60.86% of patients had severe chronic heart failure (NYHA Class III-IV), while 75.36% had vitamin D insufficiency. This high prevalence is consistent with previous studies reporting frequent vitamin D insufficiency among patients with heart failure, particularly in low- and middle-income countries [8,15,21]. Similar findings have been reported in Pakistan, where inadequate vitamin D levels are common despite abundant sunlight exposure. Factors such as inadequate dietary intake, limited outdoor activity, urban lifestyle, and poor awareness of vitamin D supplementation may contribute to this widespread deficiency [16,17]. These findings highlight vitamin D insufficiency as a common finding among patients with chronic heart failure and support further investigation into its clinical significance.

In the present study, lower serum vitamin D levels were associated with greater severity of chronic heart failure, with vitamin D insufficiency remaining independently associated with severe disease after adjustment for potential confounding factors. These findings are consistent with previous studies demonstrating an association between reduced vitamin D levels, advanced heart failure severity, impaired cardiac function, and adverse clinical outcomes [8,10,11,15]. Similar observations have been reported across different populations, with studies from the United Kingdom, Turkey, and China indicating that lower vitamin D levels are linked to poorer cardiac prognosis among patients with chronic heart failure [9,18,19]. Nevertheless, evidence regarding the clinical impact of vitamin D supplementation remains inconclusive. While some trials have demonstrated improvements in cardiac parameters, including left ventricular ejection fraction [22], others have failed to show significant clinical benefits [20,23]. These inconsistencies may be explained by variations in study design, patient characteristics, baseline vitamin D status, and supplementation protocols, emphasizing the need for further well-designed prospective studies to determine the therapeutic role of vitamin D in CHF.

Beyond vitamin D insufficiency, previous ischemic heart disease and diabetes mellitus also emerged as important factors associated with greater CHF severity. This finding is consistent with previous studies identifying diabetes and ischemic heart disease as major contributors to heart failure progression through mechanisms including accelerated atherosclerosis, endothelial dysfunction, myocardial ischemia, and adverse ventricular remodeling [7,13,14]. Although other conventional cardiovascular risk factors have been linked with heart failure severity, their impact may vary depending on underlying patient characteristics and disease profiles. These findings highlight the importance of comprehensive risk assessment and optimal management of associated metabolic and ischemic conditions in patients with CHF [10,18].

An important finding of the present study was the association between vitamin D insufficiency and poorer in-hospital clinical outcomes. Patients with vitamin D insufficiency experienced significantly longer hospital stays, higher CCU admission rates, and increased in-hospital mortality compared with patients who had normal vitamin D levels. These findings are consistent with previous studies reporting that lower vitamin D levels are associated with increased hospitalization, recurrent heart failure admissions, and higher mortality among patients with CHF [9,18,19]. However, given the observational nature of the study and the unadjusted comparison of hospital outcomes, these findings should be interpreted as associations rather than evidence of a causal or independent effect of vitamin D status.

Several biological mechanisms may explain the association between vitamin D insufficiency and greater heart failure severity. Vitamin D receptors are widely expressed in cardiomyocytes, vascular smooth muscle cells, and endothelial cells, highlighting their role in cardiovascular homeostasis. Vitamin D insufficiency may promote activation of the renin-angiotensin-aldosterone system, systemic inflammation, oxidative stress, endothelial dysfunction, myocardial fibrosis, and adverse ventricular remodeling, leading to impaired cardiac contractility and progressive heart failure. It has also been linked to insulin resistance and metabolic dysfunction, which may further accelerate cardiovascular disease progression [12-15]. These mechanisms provide a plausible explanation for the lower LVEF and greater heart failure severity observed in the present study.

From a clinical perspective, these findings suggest that vitamin D status may represent a potential marker associated with chronic heart failure severity. However, the present study does not establish that serum vitamin D assessment improves risk stratification beyond established clinical and echocardiographic parameters. Therefore, the role of vitamin D measurement as an adjunctive tool in the evaluation or prognostic assessment of chronic heart failure requires further validation in larger prospective studies. This may be particularly relevant in resource-limited healthcare settings, where vitamin D insufficiency is common, and testing is relatively accessible.

The present study has several strengths, including the inclusion of detailed clinical, laboratory, and echocardiographic data, evaluation of both heart failure severity and in-hospital outcomes, and adjustment for relevant confounding factors in the analysis of the primary outcome. These strengths provide a comprehensive assessment of the relationship between serum vitamin D status and chronic heart failure severity.

Nevertheless, several limitations should be acknowledged. First, the retrospective single-center design limits causal inference and generalizability. Second, requiring available vitamin D, NYHA, and LVEF data may have introduced selection bias. Third, single-time-point vitamin D measurement may not reflect long-term status, and retrospective NYHA assessment during hospitalization may not represent stable functional status. Residual confounding from unmeasured factors, including nutritional status, body mass index, renal function, inflammatory status, heart failure therapy, disease duration, dietary and lifestyle factors, seasonal variation, and socioeconomic status, cannot be excluded. Formal assessment of multicollinearity was not performed. Heart failure phenotype and underlying etiology were also not consistently available. In-hospital outcomes were unadjusted and may partly reflect differences in disease severity. Future prospective multicenter studies with comprehensive adjustment for relevant confounders are warranted to confirm these findings and clarify the potential prognostic value of vitamin D.

Despite these limitations, this study provides important evidence that vitamin D insufficiency is independently associated with greater chronic heart failure severity and poorer in-hospital outcomes in a Pakistani population. Incorporating serum vitamin D assessment into routine clinical evaluation may complement conventional clinical and echocardiographic risk stratification, facilitate individualized patient management, and help identify patients with greater disease severity and poorer clinical outcomes. Prospective multicenter studies and randomized controlled trials are warranted to determine whether correction of vitamin D insufficiency improves clinical outcomes in patients with chronic heart failure.

## Conclusions

This study demonstrated an association between lower serum vitamin D levels and greater severity of chronic heart failure. Vitamin D insufficiency remained independently associated with severe heart failure after adjustment for the variables included in the multivariable model, while previous ischemic heart disease and diabetes mellitus were also associated with severe disease. Vitamin D insufficiency was also associated with longer hospital stay, higher coronary care unit admission rates, and greater in-hospital mortality; however, these outcomes were based on unadjusted comparisons and may have been influenced by differences in disease severity and other unmeasured factors. Given the retrospective observational design, these findings should be interpreted as associations and do not establish a causal or definitive prognostic role for vitamin D. Prospective multicenter studies are warranted to confirm these findings and clarify the potential clinical significance of vitamin D status in chronic heart failure.
